# Supplementary figures and images for: Equine synovial fluid small non-coding RNA signatures in early osteoarthritis
Source: BMC Vet Res. 2021 Jan 9;17:26. doi: 10.1186/s12917-020-02707-7 (PMC7796526; doi:10.1186/s12917-020-02707-7)

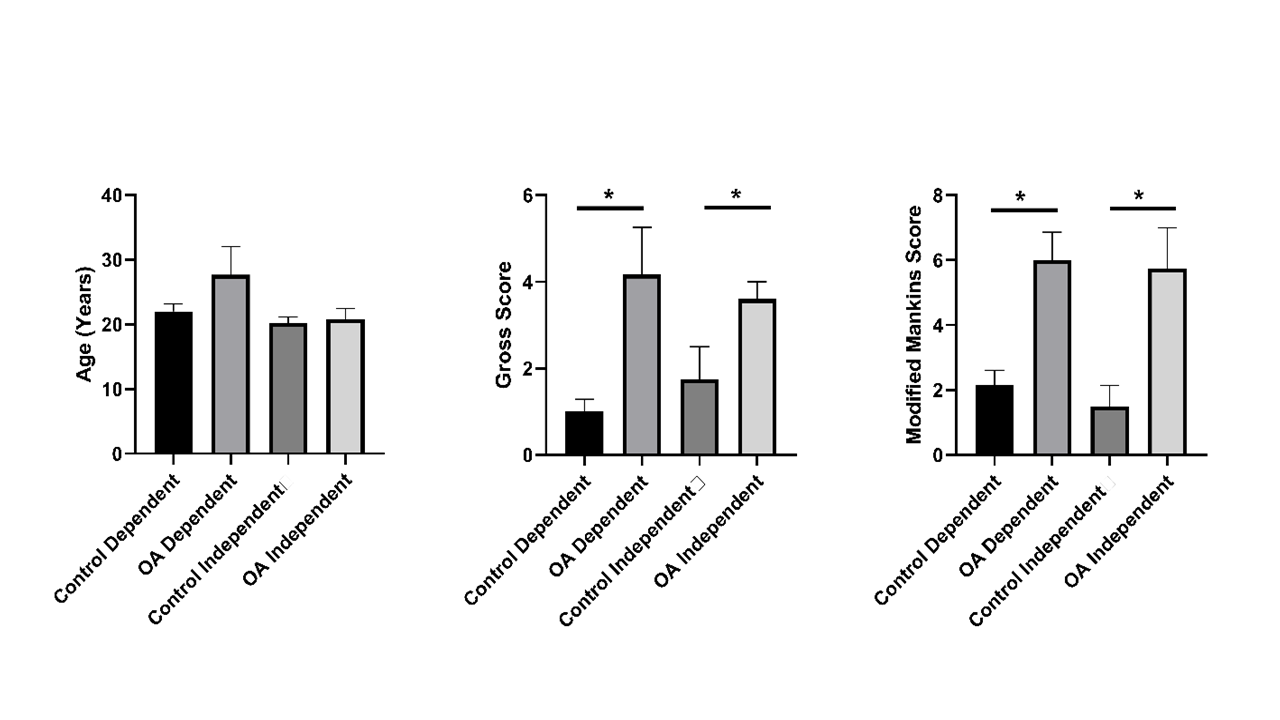

Supplement: Supplementary file 1 — Additional file 1. Histograms of age, gross score and Modified Mankin’s Score for dependent and independent equine donor cohorts (.tiff). Expressions are means and error bars ± standard error means. Statistical analysis undertaken in GraphPad Prism 8.0 using a Mann Whitney Test. P values *; P < 0.05. [file 12917_2020_2707_MOESM1_ESM.tif]
